# RETRACTED: Cheng et al. Using Comorbidity Pattern Analysis to Detect Reliable Methylated Genes in Colorectal Cancer Verified by Stool DNA Test. *Genes* 2021, *12*, 1539

**DOI:** 10.3390/genes15060805

**Published:** 2024-06-19

**Authors:** Yi-Chiao Cheng, Po-Hsien Wu, Yen-Ju Chen, Cing-Han Yang, Jhen-Li Huang, Yu-Ching Chou, Pi-Kai Chang, Chia-Cheng Wen, Shu-Wen Jao, Hsin-Hui Huang, Yi-Hsuan Tsai, Tun-Wen Pai

**Affiliations:** 1 Division of Colon and Rectal Surgery, Department of Surgery, Tri-Service General Hospital, National Defense Medical Center, Taipei 11490, Taiwan; ndmcjoe@mail.ndmctsgh.edu.tw (Y.-C.C.); wupohsien@office365.ndmctsgh.edu.tw (P.-H.W.); pencil8850@hotmail.com (P.-K.C.); wjason@mail2000.com.tw (C.-C.W.); jaosw@ndmctsgh.edu.tw (S.-W.J.); 2 Department of Computer Science and Information Engineering, National Taipei University of Technology, Taipei 10608, Taiwan; campyenju@gmail.com (Y.-J.C.); t109598090@ntut.edu.tw (Y.-H.T.); 3 Department of Computer Science and Engineering, National Taiwan Ocean University, Keelung 20224, Taiwan; gblab@email.ntou.edu.tw (C.-H.Y.); 20889002@mail.ntou.edu.tw (J.-L.H.); 4 School of Public Health, National Defense Medical Center, Taipei 11490, Taiwan; trishow@mail.ndmctsgh.edu.tw; 5 Department of Biochemistry and Molecular Cell Biology, School of Medicine, Taipei Medical University, Taipei 11042, Taiwan; toe3273917@outlook.com

The *Genes* journal retracts the article “Using Comorbidity Pattern Analysis to Detect Reliable Methylated Genes in Colorectal Cancer Verified by Stool DNA Test” [1] cited above.

Following publication, concerns were brought to the attention of the publisher regarding issues with reproducibility, scientific validity of the findings and data ownership of the list of genes discussed in ref. [1].

Adhering to our complaint’s procedure, an investigation was conducted that confirmed significant issues with the reproducibility and ownership of the data, which bring the reliability of the overall findings into question. As a result, the Editorial Board and Editor-in-Chief have lost confidence in the findings and have decided to retract this article [1].

This retraction was approved by the Editorial Board Member and Editor-in-Chief of the journal.

The authors did not agree to this retraction.
